# Impaired Barrier Function and Autoantibody Generation in Malnutrition Enteropathy in Zambia

**DOI:** 10.1016/j.ebiom.2017.07.017

**Published:** 2017-07-19

**Authors:** Beatrice Amadi, Ellen Besa, Kanekwa Zyambo, Patrick Kaonga, John Louis-Auguste, Kanta Chandwe, Phillip I. Tarr, Donna M. Denno, James P. Nataro, William Faubion, Anne Sailer, Sunil Yeruva, Tricia Brantner, Joseph Murray, Andrew J. Prendergast, Jerrold R. Turner, Paul Kelly

**Affiliations:** a Department of Paediatrics, University of Zambia School of Medicine, Nationalist Road, Lusaka, Zambia; b Tropical Gastroenterology and Nutrition Group, University of Zambia School of Medicine, Nationalist Road, Lusaka, Zambia; c Blizard Institute, Barts & The London School of Medicine, Queen Mary University of London, London, UK; d Department of Pediatrics, Washington University School of Medicine, St Louis, MO, USA; e Department of Pediatrics, University of Washington, Seattle, USA; f Department of Pediatrics, University of Virginia School of Medicine, Charlottesville, USA; g Mayo Clinic, Rochester, MN, USA; h Departments of Pathology and Medicine, Brigham and Women's Hospital and Harvard Medical School, Boston, MA, USA

**Keywords:** Enteropathy, Environmental enteric dysfunction, HIV, Malnutrition, Glucagon-like peptide 2, Microbial translocation, Autoantibodies, Tissue transglutaminase serology, deamidated gliadin peptide serology

## Abstract

Intestinal damage in malnutrition constitutes a threat to the survival of many thousands of children globally. We studied children in Lusaka, Zambia, with severe acute malnutrition (SAM) and persistent diarrhea using endoscopy, biopsy and analysis of markers and protective proteins in blood and intestinal secretions. We carried out parallel investigations in apparently healthy adults, and analyzed biomarkers only in apparently healthy children. Villus height and crypt depth did not differ in children with SAM and adult controls, but epithelial surface was reduced in children with SAM (median 445, interquartile range (IQR) 388, 562 μm per 100 μm muscularis mucosae) compared to adults (578, IQR 465,709; *P* = 0.004). Histological lesions and disruptions of claudin-4 and E-cadherin were most pronounced in children with SAM. Circulating lipopolysaccharide, a marker of bacterial translocation, was higher in malnourished children (251, IQR 110,460 EU/ml) than in healthy children (51, IQR 0,111; *P* = 0.0001). Other translocation markers showed similar patterns. Anti-Deamidated Gliadin Peptide IgG concentrations, although within the normal range, were higher in children with SAM (median 2.7 U/ml, IQR 1.5–8.6) than in adults (1.6, 1.4–2.1; *P* = 0.005), and were inversely correlated with villus height (ρ = − 0.79, *n* = 13, *P* = 0.001). Malnutrition enteropathy is associated with intestinal barrier failure and immune dysregulation.

## Highlights

• Enteropathy in children with severe acute malnutrition is characterized by variable villus blunting and epithelial disruption.
• Biomarkers indicate very high levels of microbial translocation from intestinal lumen to systemic circulation.
• Coeliac-type autoantibodies were associated with villus blunting, microbial translocation markers and mortality.

There is abundant evidence that childhood malnutrition is a major contributor to mortality in many low and middle income countries. Mortality remains unacceptably high. Here we show that severe acute malnutrition was characterized by very high levels of microbial translocation and inflammation, and severe mucosal damage with disturbed tight junction protein expression. Antibodies to tissue transglutaminase and deamidated gliadin peptides, although within the normal range, correlated with villus morphology, translocation markers and death. Malnutrition enteropathy permits mucosal inflammation, microbial translocation and immune dysregulation.

## 1 Introduction

Malnutrition underlies 45% of child deaths in low- and middle-income countries (LMICs) (McDonald et al., 2013). Severe acute malnutrition (SAM) is characterized by low weight for height (wasting), with or without edema, and affects approximately 20 million children annually. While community management of acute malnutrition has transformed outcomes for children with uncomplicated SAM (Amadi et al., 2016, Collins et al., 2006), children with medical complications such as acute infections still require hospitalization. Despite standardized treatment protocols, inpatient SAM case fatality rates remain 10–30% (Ahmed et al., 1999, Attia et al., 2016, Tickell and Denno, 2016), and are even higher among those with HIV infection (Kerac et al., 2014, Fergusson and Tomkins, 2009).

Stunting (i.e. poor linear growth) affects one-third of children in developing countries and underlies 14–17% of under-5 mortality (Olofin et al., 2013). Stunting hinders developmental potential and human capital due to its long-term impact on cognitive function and economic productivity (Prendergast et al., 2014), and resists current interventions. Implementing the ten best evidence-based nutrition-specific interventions at 90% coverage would reduce stunting by only 20%, thereby achieving only half the World Health Assembly goal of a 40% reduction by 2025 (Bhutta et al., 2013).

There is increasing recognition that gut structure and function are almost universally abnormal among children living in LMICs. Environmental enteropathy (EE) is an asymptomatic disorder which was originally described as ‘tropical enteropathy’ (Prendergast and Kelly, 2012, Keusch et al., 2014). The refractoriness of stunting to nutrition-specific interventions, together with evidence of increased intestinal permeability related to poor sanitation (Humphrey, 2009), and the impact of enteropathogens (Kosek and the MAL-ED Network Investigators, 2017) suggests that environmental enteropathy contributes to stunting (Prendergast et al., 2014, Prendergast and Kelly, 2012). Children with SAM and persistent diarrhea have a severe enteropathy, with very high burdens of intestinal infection (Amadi et al., 2001, Salazar-Lindo et al., 2003), enteric inflammation (Sullivan et al., 1991), deranged glycosylation (Amadi et al., 2009) and a T-cell activation enteropathy (Campbell et al., 2003, Veitch et al., 2001).

Among children in LMICs, enteropathy is frequently caused by a combination of EE, SAM and HIV infection (Prendergast and Kelly, 2012). A precise understanding of the pathology underlying these conditions is lacking because most studies rely on markers of gut injury as measured in blood and stool, and there have been very few studies informed by small intestinal biopsy. Using confocal laser endomicroscopy in adults (Kelly et al., 2016), we recently reported that the small intestinal mucosa has increased permeability in EE and that diminished barrier integrity is associated with microbial translocation (Brenchley and Douek, 2012, Marchetti et al., 2013). However, no studies have yet directly compared gut structure and function in children with SAM to children with only background EE using small intestinal biopsy and non-invasive biomarkers. Furthermore, the contribution of HIV infection to these enteropathies is unclear.

Here we report a cross-sectional analysis of enteropathy in children hospitalised with complicated SAM, children from a poor community with a high prevalence of stunting but without wasting, and adults from the same community. Its aim was to better understand the pathology of enteropathy in SAM and the contribution, if any, of HIV to its severity.

## 2 Methods

This study was granted approval by the University of Zambia Biomedical Research Ethics Committee (ref 006-01-13) on 11th April 2013, and was conducted in full compliance with the Declaration of Helsinki. Children hospitalised with SAM in the University Teaching Hospital were enrolled in the study following signed informed caregiver consent, on the basis that small intestinal biopsy was indicated for malnutrition and persistent diarrhea of unknown aetiology (stool parasitology and culture negative). Persistent diarrhea was defined as 3 or more loose or watery stools per day for 14 days or more. These children were all undergoing standard nutritional rehabilitation following WHO guidelines (Ashworth et al., 2003) so they were all receiving milk-based feeds (F75 and F100). HIV testing, following national guidelines, was carried out as part of their routine care on the ward, using serology (Unigold and Determine) and with PCR confirmation for children under 18 months of age. HIV infected children were started on antiretroviral therapy before discharge from the ward. Adult volunteers were recruited in Misisi, Lusaka, using a 3-stage consent process as previously described (Zulu et al., 2014). Children in the community were identified during the course of a community malnutrition screening programme (Amadi et al., 2016) and caregivers provided written informed consent to join the study. Adults and children from the community were screened for recent episodes of diarrhea (one month), NSAID use (one month), antibiotic use (one month) and excluded and treated if needed. Thus, 20 adults were excluded because of concurrent illness, pregnancy or use of antibiotics or NSAIDs, so 61 adults were studied, and of these 41 adults had satisfactory biopsies for morphometry.

### 2.1 Clinical Procedures - Adults

In adult participants, following an overnight fast, blood and urine samples were collected and then 100 ml of test solution A was given by mouth. After exactly 3 h, further urine and blood samples were collected. 1 ml of 20% chlorhexidine was added to urine samples. Blood samples were collected into EDTA, plain and trace element-free lithium heparin tubes (Vacutainer, Becton Dickinson; and TekLab respectively). Blood samples were kept in the dark for 20 min then centrifuged (537*g*, 15 min). Test solution A contained 1 g l-rhamnose (Sigma, Poole, UK), 5 g lactulose (as lactulose syrup USP) per dose, diluted in water to 100 ml.

The next day, after an overnight fast, urine and blood samples were collected as above. After 2–3 min of observation and oxygen saturation recording, intravenous sedation (2.5–10 mg diazepam, 50–100 mg pethidine) was given and endoscopy performed using Pentax EG2990i gastroscopes (Pentax, Slough, UK). 2 ml of gastric contents were aspirated from the stomach for pH measurement, and 0.5–3 ml intestinal fluid was aspirated slowly from the duodenal lumen. Three biopsies were collected into normal saline, orientated under the dissecting microscope (Swift Optical Instruments, Schertz, TX at 10 × magnification) in the endoscopy unit, the villus configuration noted, and the biopsies fixed in formal saline. After 3 h, further blood and urine samples were collected, and the exact time noted.

### 2.2 Endoscopy (Children)

Children were evaluated on the day of endoscopy by an experienced paediatrician (BCA) to confirm fitness for endoscopy, and then fasted from 0600 after a feed of F100, until 1000, the time of the endoscopy. Endoscopy was carried out using a Pentax 2490k paediatric gastroscope (8 mm external diameter) under sedation by an anaesthetist using ketamine-based protocols. Two biopsies were orientated and fixed as above. Lactase deficiency was tested in an additional biopsy for using the Biohit Lactose Intolerance Quick Test kit (Biohit, Ellesmere Port, UK). Test solution C, for children, contained 0.2 g rhamnose and 1 g lactulose per dose, made up to 50 ml, and was instilled down the endoscope as previously described (Kelly et al., 2004).

### 2.3 Clinical Procedures (Children in the Community)

Absorption and permeability testing was carried out using a very similar protocol to that used in adults, except that the period of fasting was limited to 1 h and that test solution C was taken orally. HIV testing was not carried out on these children.

### 2.4 Morphometry

Biopsies were processed, paraffin embedded, sectioned into 3 μm sections and stained with haematoxylin and eosin. Morphometry was carried out using NanoZoomer Digital Pathology (Hamamatsu Photonics, Hamamatsu, Japan) as previously described (Kelly et al., 2004, Kelly et al., 2016) (Supplementary Fig. S1). The following measurements were made: villus height (VH) and width (VW), crypt depth (CD), villus mucosal perimeter (VP) as a measure of epithelial surface area and related to length of muscularis mucosae, and villus cross-sectional area (VA) as a measure of villus volume. VH/CD and villus surface area/volume ratio (SAVR) (Kelly et al., 2016) were derived from these. Claudin-4 immunostaining was performed on 3 μm sections (see Supplementary material).

### 2.5 Sugar Analysis

Urine samples were collected at 3 h in adults, and 60 min in children. Lactulose and rhamnose were assayed by mass spectrometry in urine samples as previously described (Faubion et al., 2016).

### 2.6 Biomarkers

To estimate the severity of translocation, we measured plasma lipopolysaccharide (LPS) and carried out PCR for bacterial 16S ribosomal DNA as direct markers of translocation (see Supplementary data), while serum lipopolysaccharide binding protein (LBP), soluble CD14 (sCD14), CD163 and C-reactive protein (CRP) were used as indirect markers of the host response to translocated bacterial molecules (Table 2). Quantification of LPS was by the Pyrochrome Limulus Amoebocyte Lysate (LAL) assay (Associates of Cape Cod, Liverpool, UK). Serum LPS binding protein, CRP, sCD14, CD163, IGF-1 and IGFBP3 were measured by ELISA (R&D systems, Abingdon, UK: RRID:SCR_006140). DNA was isolated from plasma using the QIAamp DNA Mini Kit (Qiagen: RRID:SCR_008539), according to the manufacturer's instructions, then analyzed by PCR (see Supplementary material). Total immunoreactive GLP-2 was measured in fasted serum by ELISA (Millipore, St Charles, MO: RRID:SCR_008983). Intestinal FABP was measured by ELISA (Hycult, Cambridge Bioscience, Cambridge, UK: RRID: SCR_002245). Protein was extracted from duodenal aspirates using a lysis buffer made up of 2-mercaptoethanol and Laemmli buffer, then Western blots were probed for TFF3 using mouse monoclonal antibody to TFF3 (Abcam, Cambridge, UK: RRID: SCR_012931) and detected using VECTASTAIN anti-mouse IgG Biotinylated antibody (Vector laboratories, Peterborough, UK: RRID:SCR_000821). All assays were conducted in duplicate.

### 2.7 Coeliac Serology

Tissue transglutaminase IgA antibodies were measured in serum using the Orgentech ELISA kit (Launch Diagnostics, Longfield, UK) and the Quanta Lite ELISA kit (Inova Diagnostics, San Diego, USA). Antibodies to deamidated gliadin peptides were measured using the Quanta Lite Gliadin IgGII kit (Inova). The Orgentech ELISAs were run in both the Lusaka and Mayo Clinic laboratories (ρ = 0.88; *p* < 0.0001); the Inova TTG and DGP ELISAs were run only in the Mayo Clinic.

### 2.8 Data Analysis

Data analysis was carried out using Stata 13 (Stata Corp, College Station, TX). Spearman's rank correlation coefficient and the Kruskal-Wallis test were used for hypothesis testing as most variables were not normally distributed.

### 2.9 Role of the Funding Source

The funding sources played no role in the decision to publish, data analysis, or in the drafting of the manuscript. The corresponding author had free access to all data.

## 3 Results

We enrolled 34 children with SAM and persistent diarrhea not responsive to standard treatment (Supplementary Table 1). Three of these children died later during hospitalization. During a programme of screening for malnutrition in Misisi, 101 children who were not acutely malnourished were also evaluated (Supplementary Table 1). Written consent was obtained from caregivers for child participants. Following a 3-stage recruitment and consent process, 81 unselected HIV-seropositive and HIV-seronegative adults from Misisi agreed to participate in the study (Supplementary Table 2).

### 3.1 Morphological Severity of Enteropathy

**Fig. 1:**
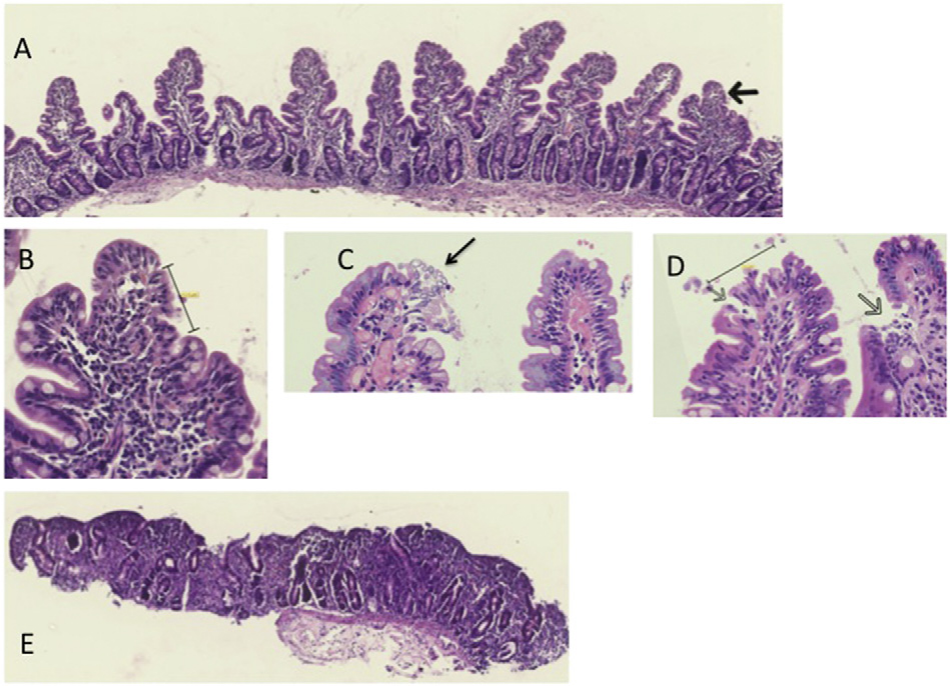
Mucosal histology in severe acute malnutrition. A, histological section from a child with severe acute malnutrition (SAM) showing moderate villus blunting (villus height 247 μm). B shows a higher magnification of A at the point of the arrow, an early epithelial discontinuity measuring 62.5 μm. C and D, detachment of pale and irregular epithelial cells in biopsies from two adults with EE; at the site of these more extensive discontinuities or microerosions (black arrows) the basement membrane is exposed to the luminal stream. E, a biopsy from a child with SAM showing subtotal villus atrophy.

**Fig. 2:**
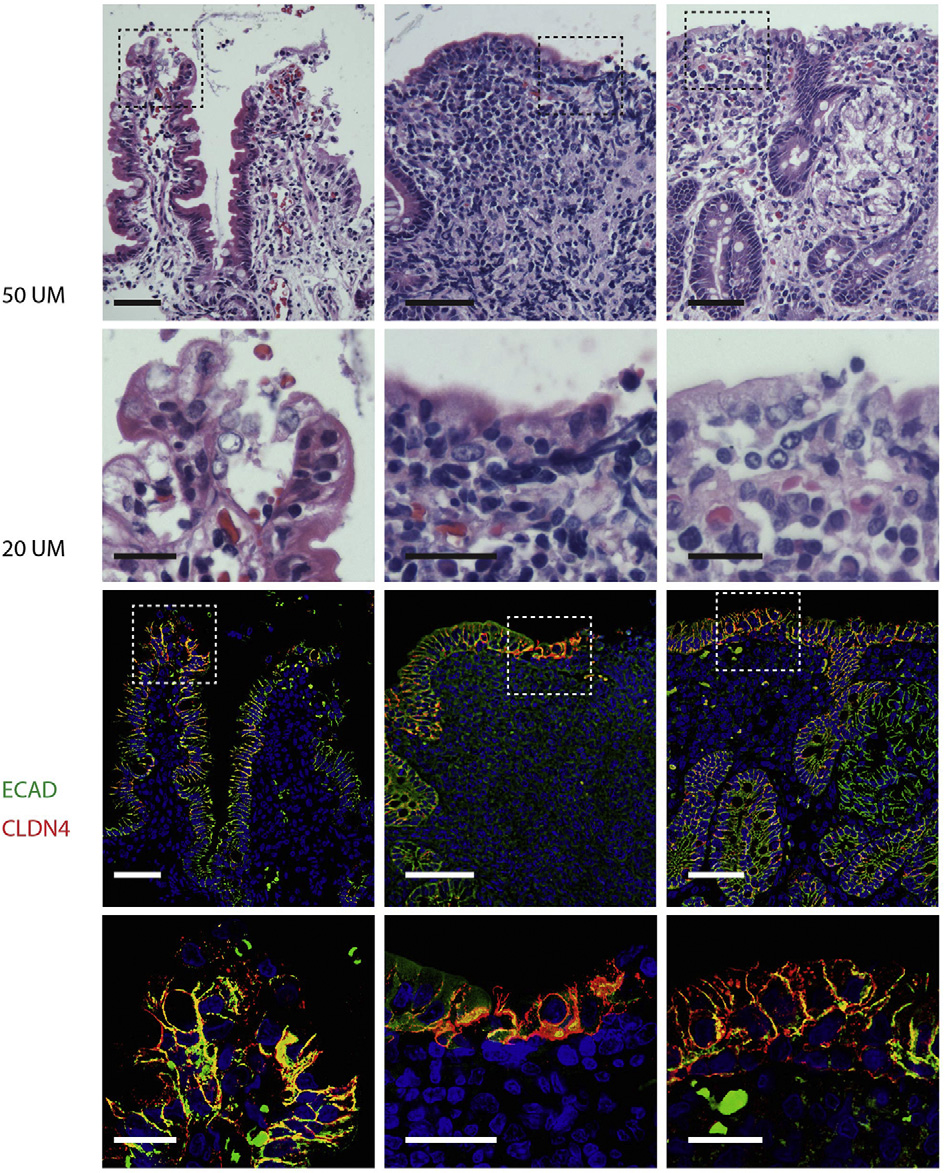
Epithelial histology and disruption of claudin 4 and E-cadherin. Epithelial breaks imaged in pairs in nearby sections from three children with severe acute malnutrition, each in a separate column. Top row: haematoxylin/eosin (H&E) stains with scale bar representing 50 μm. Second row, magnified H&E images of boxes from the first row with scale bars representing 20 μm. Third row, images of same sections shown in first row, with E-cadherin immunostaining in green, claudin-4 immunostaining in red, and nuclei (DAPI) in blue; scale bars at 50 μm. Fourth row, further magnification of boxes from third row, using the same colour code, and scale bars at 20 μm. (For interpretation of the references to colour in this figure legend, the reader is referred to the web version of this article.)

Children hospitalised with SAM, and all adults, underwent endoscopy to collect duodenal biopsies. Children from the community did not have endoscopy. All biopsies were examined immediately after endoscopy using a binocular microscope and all demonstrated evidence of villus morphological change, with ridges and convolutions and absence of finger-like villi. This corresponded to histological evidence of villus blunting and infiltration of the mucosa by chronic inflammatory cells (Fig. 1). Villus height (VH), crypt depth (CD), villus width (VW), and VH:CD ratio were measured satisfactorily in biopsies from 41 adults and 22 children; they did not differ significantly between adults with EE and children with SAM, irrespective of HIV status (Table 1). However, two biopsies from children with SAM showed total villus atrophy (Fig. 1); both these children died during their admission. One other child died on the ward; this child had partial villus atrophy with a measured villus height (VH) of 198 μm and a villus height:crypt depth ratio of 1.2. Overall, the three children who died in hospital had lower VH (median 83 μm; IQR 74–198) than those who survived (216 μm; 175–246; *P* = 0.04) but crypt depth (CD) did not differ (170 vs 156 μm; *P* = 0.32). SAVR was reduced in HIV-infected children with SAM, but otherwise HIV status had little impact on mucosal morphology (Table 1). Epithelial breaks (Fig. 1, Fig. 2) were seen in all biopsies from children with SAM and in 53/54 (98%) of 54 (98%) adults evaluated. The frequency of breaks was unrelated to villus height, crypt depth or other morphometric parameters (data not shown).

**Table 1:** Morphometry and gastric pH in adults and children.

Measurement	HIV negative SAM (*n* = 14)	HIV positive SAM (*n* = 8)	HIV negative adults (*n* = 31)	HIV positive adults (*n* = 10)	*P* (all groups)
Villus height (μm)	211 (164–246) [74–281]	206 (180–239) [83–284]	217 (196–248) [151–362]	242 (204–280) [195–293]	0.12
Crypt depth (μm)	155 (144–170) [106–208]	173 (144–200) [126–220]	161 (144–174) [128–218]	170 (136–195) [123–208]	0.80
Villus width (μm)	143 (129–166) [93–501]	175 (163–247) [126–524]	171 (147–195) [103–252]	189 (145–227) [116–261]	0.58
Villus epithelial surface (μm)	467 (367–614) [132–930]	426 (407–499) [139–562]	634 (465–714) [273–789]	522 (462–562) [290–756]	0.005
Villus cross-sectional area (μm^2^) per 100 μm muscularis mucosae	15,006 (11008–18,111) [5510–32,179]	16,505 (13909–19,363) [7481–23,604]	18,726 (16489–25,083) [13702–31,595]	19,371 (14679–21,437) [13613–25,515]	0.02
VH:CD	1.37 (0.96–1.56) [0.44–1.89]	1.30 (1.12–1.46) 0.41–1.53]	1.35 (1.22–1.51) [0.89–2.25]	1.44 (1.35–1.57) [1.21–1.84]	0.35
Villus SA:volume ratio	0.033 (0.029–0.035) [0.020–0.040]	0.028 (0.021–0.029) [0.018–0.030]	0.031 (0.027–0.035) [0.013–0.044]	0.028 (0.026–0.031) [0.020–0.034]	0.61
Gastric pH	3.5 (2.0–5.5) *n* = *15*	4.8 (3.0–6.0) *n* = 10	1.5 (1.0–2.5)	4.0 (1.5–5.5)	0.0007
Hypochlorhydria (fasting pH > 4.0)	7/15 (47%)	6/10 (60%)	5/35 (14%)	10/21 (48%)	0.05
Lactase deficiency	6/13 (46%)	1/4 (25%)	nt	nt	

Values given are median with interquartile range in parentheses () and range in brackets []. SAM, severe acute malnutrition; VH, villus height; CD, crypt depth; SA, surface area; nt, not tested.

Of 17 children with SAM from whom a biopsy was available for lactase testing, 7 (41%) were lactase deficient (Table 1). Villus height was markedly reduced in 3 children with lactase deficiency whose morphometry data were available (median 83 μm; IQR 74–143) compared to 8 children without lactase deficiency (median 201 μm; IQR 170–230; *P* = 0.02).

### 3.2 Claudin 4 and E-Cadherin Immunostaining

To clarify the significance of these breaks, we performed immunostaining for the tight junction protein claudin-4 and the adherens junction protein E-cadherin (Fig. 2). Overall, claudin-4 expression was reduced in all biopsies, compared to previous experience in healthy tissue (Turner, 2009, Gunzel and Yu, 2013), and E-cadherin immunostaining was disrupted at the sites of epithelial breaks. However, claudin-4 immunostaining was markedly increased and disorganised at the villus tip and at sites of epithelial breaks (Fig. 2).

### 3.3 Biomarkers of Mucosal Integrity

Lactulose and rhamnose, measured as percentage excretion of the oral dose given and as a urinary ratio, were used to measure intestinal permeability (Faubion et al., 2016). Failed tests occurred in 65% of the children with SAM and 30% of the children in the community; this was largely due to inability to pass urine in the 60 min specified in the protocol. Lactulose recovery and the lactulose:rhamnose (LR) recovery ratio were greater in malnourished children than in adults or community children (Table 2). The LR ratio was inversely correlated with villus surface area in adults and children in whom both morphometric measurements and satisfactory LR measurements were obtained (ρ = − 0.35, *P* = 0.02; *n* = 47), but with no other morphometric measurement. Rhamnose recovery was positively associated with villus surface area:volume ratio (ρ = − 0.40, *P* = 0.005; *n* = 47) but not with other morphometric parameters. Intestinal type fatty acid binding protein (FABP) was increased in all three groups of children, especially children with SAM, compared to adults (Table 2).

**Table 2:** Biomarkers of microbial translocation and mucosal dysfunction in environmental, HIV and malnutrition enteropathy.

Biomarker	Normal range^a^	HIV negative children with SAM (*n* = 20)	HIV positive children with SAM (*n* = 14)	HIV negative adults (*n* = 39)	HIV positive adults (*n* = 22)	Community children (*n* = 101)	*P*
16S rDNA PCR positive	Not established	8/14 (57%)	4/6 (67%)	19/34 (56%)	14/20 (70%)	13/82 (16%)	< 0.0001
LPS (EU/ml)	Not established	272 (142–600) [0–2097] *n* = 15	120 (0 − 223) [0–339] *n* = 7	187 (92–374) [23–1219] *n* = 36	122 (66–202) [36–536] *n* = 22	50.9 (0 − 111) [0–869] *n* = 108	0.0001
LBP (ng/ml)	Not established	150 (148–190) [135–258] *n* = 15	253 (170–356) [159–530] *n* = 7	22.9 (19.8,28.5) [15.1–35.9] *n* = 39	28.9 (26.6,37.2) [15.5 = 43.1] *n* = 22	40.3 (32.9–48.2) [1.8–82.8] *n* = 94	0.0001
sCD14 (mg/l)	0.8–3.2	2.53 (2.19–3.31) [1.10–4.51] *n* = 16	2.84 (1.50–3.72) [1.04–5.49] *n* = 7	1.62 (1.34–1.84) [0.74–2.46] *n* = 39	1.84 (1.40–2.41) [0.54–4.24] *n* = 22	1.78 (1.54–2.14) [0.80–3.37] *n* = 78	0.0001
CD163 (μg/l)	88–902	1226 (1007–1983) [425–3860] *n* = 16	1482 (661–3147) [551–3441] *n* = 7	618 (408–804) [188–1706] *n* = 39	684 (453–1068) [50–2605] *n* = 22	874 (686–1305) [318–2331] *n* = 78	0.0001
CRP (mg/l)	0–5.0	1.75 (0.63–5.09) [0.05–21.0] *n* = 16	3.67 (0.50–10.31) [0.46–21.0] *n* = 7	0.95 (0.47–2.45) [0–16.4] *n* = 39	2.31 (0.64–10.3) [0.14–21.0] *n* = 22	nt	ns
FABP (ng/ml)	Not established	3.14 (2.5–4.8) [0.38–13.5] *n* = 19	4.52 (2.76–13.47) [2.66–15.8] *n* = 7	0.65 (0.44–0.86) [0.18–3.2] *n* = 39	0.64 (0.49–1.4) [0–3.43] *n* = 22	2.01 (1.33,3.04) [0.17–13.2] *n* = 74	0.0001
GLP-2 (ng/ml)	Median 11.6, IQR 7.0–18.6	1.8 (1.0–2.3) [0.5–5.1] *n* = 14	4.1 (2.2–5.6) [0.4–7.6] *n* = 7	1.2 (0.8–2.1) [0–5.4] *n* = 39	1.0 (0–2.0) [0–5.3] *n* = 22	2.0 (1.5–2.7) [0–5.4] *n* = 75	0.0007
IGF-1 (ng/ml)	43–182	11.7 (9.8–20.2) [9.3–39.4] *n* = 15	9.3 (9.3–10.1) [9.3–12.7] *n* = 6	nt	nt	26.1 (19.4–36.4) [0–143] *n* = 79	0.0001
IGFBP-3 (μg/ml)	1.53–3.09	0.89 (0.35–1.67) [0.23–2.25] *n* = 15	0.70 (0.49–0.89) [0–1.34] *n* = 6	nt	nt	1.22 (0.93–1.43) [0.34–2.53] *n* = 76	0.02
Lactulose recovery (% of administered dose)	Children: median 0.01 (range 0–0.07) Adults: mean 0.281 (sd 0.014)	0.0287 (0.017–0.083) [0.002–0.18] *n* = 8	0.10 (0.017–0.474) [0.015–0.78] *n* = 4	0.0823 (0.049–0.120) [0.0147–0.413] *n* = 38	0.0597 (0.04–0.137) [0.0009–0.25] *n* = 22	0.009 (0.002–0.026) [0–0.299] *n* = 76	0.0001
Rhamnose recovery (% of dose)	Children: median 0.38 (range 0–1.84) Adults: mean 11.1 (sd 0.4)	0.087 (0.072–0.273) [0.03–1.16] *n* = 8	0.15 (0.068–0.46) [0.03–0.72] *n* = 4	1.70 (0.877–2.62) [0.3–5.66] *n* = 38	1.21 (0.42–1.85) [0.3.51] *n* = 22	0.17 (0.03–0.42) [0.007–1.75] *n* = 76	0.0001
L:R recovery ratio	Children: median 0.14 (range 0.06–1.00) Adults: mean 0.026 (sd 0.0001)	0.213 (0.129–0.289) [0.062–1.070] *n* = 8	0.762 (0.357–1.00) [0.148–1.05] *n* = 4	0.055 (0.045–0.072) [0.014–0.180] *n* = 38	0.066 (0.039–0.103) [0.030–0.50] *n* = 20	0.071 (0.037–0.12) [0–1.289] *n* = 76	0.0001
Anti-TTG IgA (Inova) (U/ml)	Below 10	0.54 (0.51–0.59) [0.48–0.72] *n* = 16	0.61 (0.56–1.09) [0.54–1.99] *n* = 7	0.57 (0.54–0.64) [0.05–3.66]	0.60 (0.55–0.63) [0.52–0.84]	nt	0.02
Anti-TTG IgA (Orgentech) (U/ml)	Below 10	2.12 (2.02–2.21) [1.93–2.84] *n* = 16	2.44 (2.30–2.68) [2.22–2.87] *n* = 4	2.30 (2.18–2.46) [2.10–6.15]	2.23 (2.18–2.36) [2.06–2.71]	0 (0–0) 0–2.81]	0.0001
Anti-DGP IgG (U/ml)	Below 30	1.6 (1.4–3.6) [1.1–12.3] *n* = 16	15.7 (4.0–45.7) [2.4–102.2] *n* = 7	1.48 (1.41–2.04) [1.20–4.60]	1.51 (1.42–1.96) [1.20–6.58]	nt	0.0007

*P* is the result of Kruskal-Wallis test or Fisher's exact test across all groups. *n* is shown where it is less than the number of participants shown at the head of the column. nt, not tested; ns, not significant. LPS, lipopolysaccharide; LBP, LPS binding protein; sCD14, soluble cluster of differentiation 14; CD163, cluster of differentiation 163; CRP, C-reactive protein; FABP, fatty acid binding protein; GLP-2, glucagon-like peptide 2; IGF-1, insulin-like growth factor-1; IGFBP-3, IGF binding protein-3; TTG, tissue transglutaminase; DGP, deamidated gliadin peptides.

a Reference ranges are obtained from package inserts in ELISA kits, except for: mono- and disaccharide excretion in children (Faubion et al., 2016), mono- and disaccharide excretion in adults (Menzies et al., 1999); GLP-2 (Hoffmann, 2009); normal values for some molecules are not established. In the study by Menzies et al. (1999) a 5-hour collection was used so recoveries would be expected to be higher; the LR ratio remains unchanged over 3 or 5 h.

### 3.4 Markers of Microbial Translocation

Lipopolysaccharide (LPS) concentrations were higher in plasma which was also positive for bacterial 16S rRNA gene DNA (median 137, IQR 61–257 EU/ml) compared to plasma in which no bacterial DNA was detected (median 79, IQR 0–191 EU/ml; *P* = 0.01). Direct markers of translocation (16S DNA and LPS) were markedly higher in adults and malnourished children than in healthy children (Table 2). Indirect markers of bacterial translocation (LBP and CD163, markers of the host response to pathogen associated molecular patterns) were elevated in malnourished children compared to other groups (Table 2). In all children combined, irrespective of nutritional status, IGF-1 was modestly and inversely correlated with LPS (ρ = − 0.23, *P* = 0.02; *n* = 100), consistent with translocation of microbes or their components being inhibitory for growth hormone axis activity. No such relationships were detected between IGF-1 and inflammatory markers (CRP, sCD14, CD163).

### 3.5 Mucosal Repair Peptides

**Fig. 3:**
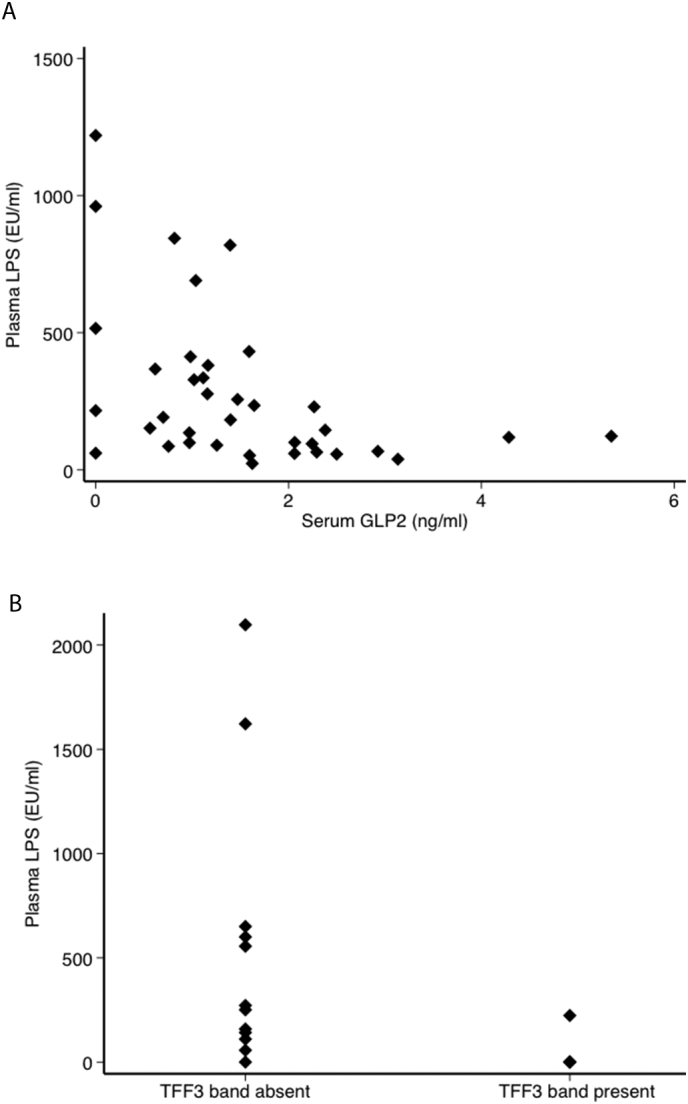
Molecules protective against microbial translocation. (A) GLP-2 in adults was negatively associated with circulating lipopolysaccharide (LPS; ρ = − 0.35; *P* = 0.01 in the whole group, but in HIV seronegatives the correlation was stronger (ρ = − 0.51; *P* = 0.001). (B) Presence of TFF3 immunoreactivity in duodenal aspirates of children was associated with greatly reduced circulating LPS (*P* = 0.03).

We measured two proteins which promote epithelial restitution: the hormone glucagon-like peptide 2 (GLP-2) (Mutanen and Pakarinen, 2016) in blood, and trefoil factor 3 (TFF3) (Hoffmann, 2009) in intestinal secretions. Serum GLP-2 concentrations were higher in children than adults (Table 2), and lower than the reference range (Mutanen and Pakarinen, 2016) in all our participants. LPS was inversely correlated with GLP-2 (ρ = − 0.35, *P* = 0.01; *n* = 54) and this association was stronger in HIV negative adults (ρ = − 0.51; *P* = 0.001; *n* = 37; Fig. 3). In children with SAM, translocation was associated with reduced TFF3: LPS was undetectable in 3 of 4 children with detectable TFF3 dimer in duodenal aspirates (*P* = 0.03; Fig. 3).

### 3.6 Anti-Tissue Transglutaminase (TTG) and Related Antibodies

**Fig. 4:**
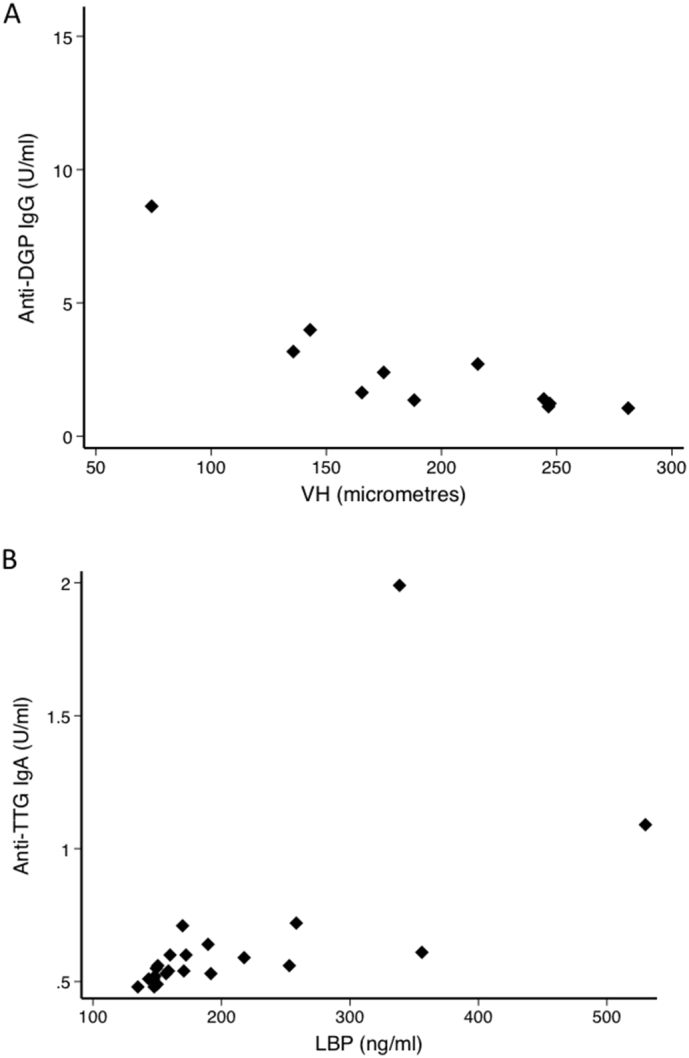
Coeliac type antibodies. Anti-deamidated gliadin peptides (DGP) and anti-tissue transglutaminase (TTG) antibodies in children. (A) Correlation between anti-DGP antibody concentration in serum and villus height (ρ = − 0.79; *P* < 0.0001); two points with anti-DGP concentrations above 40 U/ml omitted for clarity. (B) Correlation between anti-TTG antibody concentration (Inova system) in serum and circulating lipopolysaccharide binding protein, LBP (ρ = 0.83; *P* < 0.0001).

In view of the extensive structural change seen in some biopsies, and most notably in two children with subtotal villus atrophy who expired before discharge, testing for coeliac disease serology was explored in children with SAM and in adults, even though the therapeutic as well as the staple diets of children with SAM contain minimal gluten. Tissue transglutaminase (TTG) IgA antibody concentrations, by two different ELISAs, were normal in sera from all adults and children, and the results of both ELISAs correlated closely (ρ = 0.81, *P* < 0.0001; *n* = 20). However, anti-TTG antibodies, even within the normal range, were higher in HIV infected children (median 2.4 U/ml, IQR 2.3–2.7) than in HIV-uninfected children (2.1 U/ml, 2.0–2.2; *P* = 0.01). This difference was not seen in adults. IgG anti-deamidated gliadin peptide (DGP) antibodies were negative in adults, but positive in 3 children, one of whom died in hospital and one of whom died after discharge. Although TTG IgA concentrations were within the normal range, the two children who died in hospital had higher TTG IgA (Inova Quant) concentrations (0.72 and 1.09 U/ml) than children who survived (median 0.55; IQR 0.52–0.6); this was statistically significant (*P* = 0.04) even though the number of events was very small. Anti-DGP IgG concentrations were higher in children with SAM (median 2.7 U/ml, IQR 1.5–8.6) than in adults (1.6, 1.4–2.1; *P* = 0.005), and were inversely correlated with villus height (ρ = − 0.79, *n* = 13, *P* = 0.001; Fig. 4). Anti-DGP IgG concentrations were also higher in children who died (median 55 U/ml; IQR 8.6–102) than in those who did not (2.7; 1.5–5.1; *P* = 0.04). All three serological tests were strongly correlated with LBP (ρ = 0.71, *P* = 0.006 for TTG IgA (Orgentech); ρ = 0.83, *P* < 0.0001 for TTG IgA (Inova; Fig. 4); and ρ = 0.57, *P* = 0.006 for anti-DGP IgG).

## 4 Discussion

Children living in LMICs may have one or more enteropathies, with or without a contribution from HIV infection. Together they are believed to contribute substantially to child morbidity and mortality. Here we report a comparison of the pathology and severity of enteropathy in children with severe acute malnutrition and well children and adults living in a community where the prevalence of stunting is high and from which, historically, many admissions to the hospital malnutrition ward have originated. Using histopathology and other biomarkers of enteropathy, we found that the principal difference between these different groups was one of degree: severe malnutrition is associated with severe enteropathy, intense microbial translocation and severe systemic inflammation. No biopsy was normal: almost all biopsies demonstrated villus remodeling and epithelial breaks with disorganization of claudin 4 and E-cadherin. The most severe enteropathy (total villus atrophy) was associated with subsequent death in both children in whom it was found. Children with malnutrition and persistent diarrhea had the most abnormal markers of microbial translocation and inflammation. However, the severity of the villus blunting did not explain the translocation observed, as the differences in translocation and inflammation greatly outweighed the modest morphometric differences. A comparison of our data with previous morphometric studies is provided in Supplementary material. We readily acknowledge the difficulties of interpretation imposed by being unable to justify (ethically) taking biopsies from apparently healthy children with presumed environmental enteropathy. Our understanding would be greatly enhanced if well-orientated biopsy material from healthy children were available for formal morphometric analysis.

Microbial translocation is the passive movement of gut microbes from the lumen of the intestine into lymphatics, portal blood and thence into the central circulation (Brenchley and Douek, 2012, Marchetti et al., 2013). It appears to be a central derangement in the enteropathy of SAM, with extraordinarily high concentrations of both direct and indirect markers of translocation in blood. Translocation markers were associated with reduced circulating IGF-1 concentrations in children, consistent with the hypothesis that microbial translocation adversely affects child growth. The biomarkers of translocation that we measured could be the degradation products of whole bacteria which cross the mucosa, or they could reflect the translocation of only constituent molecules such as LPS and DNA; both of these act as ligands for pattern recognition receptors (Wells et al., 2017).

Having found very severe mucosal damage in two children with SAM, we were concerned to exclude coeliac disease, and conducted three different assays for coeliac disease serology in two laboratories in Zambia and the USA. While the great majority of these serological results were within the normal range, closer scrutiny revealed that in children with severe acute malnutrition, concentrations of antibodies to tissue transglutaminase and deamidated gliadin peptides were very strongly correlated with LBP (a marker of the host response to bacterial translocation), inversely correlated with villus height, and were highest in the children who subsequently died. We postulate that in the presence of mucosal T cell activation (Campbell et al., 2003, Veitch et al., 2001) and epithelial leakiness the cells of the lamina propria can generate autoantibodies, and that this autoreactivity exacerbates mucosal pathology. Autoantibody generation has been reported in Crohn's disease (Ribeiro-Cabral et al., 2011) and in olmesartan-induced enteropathy (Esteve et al., 2016) where it was reversible. Exposure to gluten and gliadin peptides is thought to be essential for development of the autoantibodies which characterize coeliac disease (Sollid and Jabri, 2013), but in Zambia the staple diet is maize, and children being treated for SAM have a therapeutic feed which includes no gluten (Ashworth et al., 2003). We have previously reported that elemental feeds enhance weight gain during nutritional rehabilitation in children with SAM^37^ (Amadi et al., 2005). These serological data suggest that coeliac antibodies, even within the clinically normal range, may be a useful biomarker of the severity of enteropathy; further work is needed in other settings to confirm or refine this hypothesis. It does not mean that these children have coeliac disease: the concentrations were within the normal range, the children were exposed to minimal gluten. There are few data on the prevalence of those HLA alleles which mediate coeliac disease in Africa (Kang et al., 2013). We have considered whether the increased anti-TTG and anti-DGP antibodies may be due to hypergammaglobulinaemia. There is good evidence that IgA concentrations are increased in SAM, particularly in the presence of oedema (Rytter et al., 2014). Unfortunately, insufficient serum was available to measure total immunoglobulins, as blood sampling volumes from these children are limited. The consistency between IgA anti-TTG and IgG anti-DGP suggests that this is not merely a reflection of increased circulating IgA concentrations, and we observed no correlation between severity of edema and antibody concentrations (data not shown).

Claudin-4 is a structural protein of the tight junction that augments epithelial barrier function *in vitro* (Turner, 2009, Gunzel and Yu, 2013) and has been characterized in vivo as a paracellular anion channel (Hou et al., 2010). Maintenance of tight junctions requires the physical apposition of adjacent cells which are held together, in part, by adherens junctions. The principal adherens junction adhesive protein E-cadherin also interacts with β-catenin to regulate Wnt signaling protein, thereby allowing loss of intercellular adhesion to promote the proliferation that is ultimately required for repair. Our data suggest marked disruption of tight junction distribution. This would be sufficient to explain the increased permeation of lactulose, but insufficient to explain the translocation of intact bacteria, if that is indeed how microbial translocation occurs.

‘HIV enteropathy’ was used at one time to describe the severe diarrhea-wasting syndrome which was so prominent in the early days of the HIV epidemic, particularly a severe pathogen-negative diarrhea-malabsorption syndrome. In populations where EE is also prevalent, HIV enteropathy is difficult to recognize as it is morphologically indistinguishable from EE apart from an increase in crypt depth, which we previously reported only in patients with advanced immunosuppression (Kelly et al., 2004). Here we report that villus surface area: volume ratio was 10% reduced in HIV infection, but no other parameters differed. HIV infection was not associated with an increased level of LPS or an increased detection of bacterial DNA in either adults or children, but it was associated with considerably increased CRP.

## Appendix A Supplementary Data

Supplementary material Image 1
