# Supplementary material for: Impaired Barrier Function and Autoantibody Generation in Malnutrition Enteropathy in Zambia
Source: eBioMedicine. 2017 Jul 19;22:191–9. doi: 10.1016/j.ebiom.2017.07.017 (PMC5552244; doi:10.1016/j.ebiom.2017.07.017)
Supplement: Supplementary file 1 — Supplementary material [file mmc1.docx]

**Supplementary material for Amadi et al: Impaired barrier function and autoantibody generation in malnutrition enteropathy in Zambia**

**Supplementary Table 1** Demographic, clinical and nutritional characteristics of child participants

|  | HIV negative SAM | HIV positive SAM | Community controls | *P*  SAM vs controls | *P*  SAM  HIV pos  vs neg |
| --- | --- | --- | --- | --- | --- |
|  | n = 20 | n=14 | n = 101 |  |  |
| Sex (M:F) | 11:9 | 8:6 | 42:59 | 0.17 | 1.00 |
| Age (months) | 15 (12-20)  [6-23] | 14.5 (12-21)  [9-23] | 21 (16-29)  [12-36] | <0.0001 | 0.96 |
| Duration of diarrhoea (days) | 21 (14-28)  [14-180] | 21 (21-35)  [14-120] | na | na | 0.23 |
| Current breastfeeding | 1 (5%) | 1 (14%) | 27 (28%) | 0.03 | 0.56 |
| Dehydration | 4 (20%) | 3 (21%) | na | - | 1.00 |
| MUAC (cm) | 11.7 (10.4-12.8)  [8.5-14.0] | 10.6 (10.3-11.2)  [7.9-15.8] | 14.4 (13.5-15.0)  [12.6-17.0] | <0.0001 | 0.20 |
| WAZ, median  (IQR) [range] | -3.2 (-3.8, -2.5)  [-6.7, 0.1] | -4.6 (-5.3, -3.3)  [-6.7, -1.5] | -1.2 (-2.0, -0.5)  [-3.6, 0.37] | <0.0001 | 0.052 |
| LAZ  (IQR) [range] | -2.4 (-3.4, -1.7)  [-6.2, 0.6] | -4.1 (-5.3, -2.1)  [-6.7, -1.5] | -2.3 (-2.9, -1.2)  [-4.9, 1.8] | <0.0001 | 0.09 |
| WLZ  (IQR) [range] | -2.6 (-3.9, -1.1)  [-5.6, -0.1] | -3.8 (-4.4, -2.5)  [-5.9, -1.0] | -0.3 (-1.1, 0.25)  [-2.3, 1.8] | <0.0001 |  |
| Stunted | 7 (35%) | 8 (57%) | 22 (22%) | 0.01 | 0.30 |
| Anti-Retroviral Therapy (ART) on admission | 0 | 2 | 0 |  |  |

SAM, severe acute malnutrition; NT, not tested; WAZ, weight-for-age z score; LAZ, length-for-age z score; WLZ, weight-for length z score. Measurements are given as median (interquartile range)[range].

**Supplementary Table 2** Demographic and clinical characteristics of adult participants

|  | HIV seronegative  (n=39) | HIV seropositive  (n=22) | *P* |
| --- | --- | --- | --- |
| Sex (M:F) | 15:24 | 3:19 | 0.05 |
| Age (years, median and IQR) | 24 (21-36) | 32.5 (28-47) | 0.002 |
| Education beyond primary | 20 (51%) | 9 (41%) | 0.59 |
| Asset score | 2 (1-4) | 3 (2-3) | 0.86 |
| Household hygiene score | 6 (5-8) | 6 (5-8) | 0.82 |
| BMI (kg/m^2^) | 22.8 (20.8-25.2) | 22.4 (20.7-27.6) | 0.85 |
| Mid upper arm circumference (cm) | 26.8 (25.0-29.3) | 27.3 (24.5-31.1) | 0.59 |
| Ever use boiled drinking water | 8 | 3 | 0.73 |
| Ever use chlorinated drinking water | 23 | 15 | 0.59 |
| Current smoker | 2 | 2 | 0.62 |
| Currently takes any alcohol | 14 | 10 | 0.59 |
| Anti-Retroviral Therapy (ART)^a^ | na | 8/22 (36%) | na |
| CD4 | na | 442 (250-563)  [160-883] | na |
| CD4 below 350 cells/μl | na | 8/18 (44%) | na |

^a^At the time of the study, ART was made available to HIV-infected adults with CD4 cell counts of 350 cells/μl or less.

**Mucosal morphometry**

In order to obtain well-orientated histological sections, which is a prerequisite for morphometry, all biopsies were placed in normal saline immediately on collection. Within 10 minutes biopsies were transferred to a clean Petri dish and orientated under a binocular microscope (Swift Optical Instruments, Schetz, TX) at a magnification of 10x, while continuously bathed in saline so that villus structures were uppermost, then slid carefully onto cellulose acetate paper (Sartorius, Germany) and placed into formalin-saline. After several days-weeks of fixation, biopsies were placed flat (villi uppermost) in histological cartridges prior to embedding in wax and sectioning perpendicular to the paper, with the plane of cutting aligned to the crypt-villus axis. Sections were photographed using the NanoZoomer tool (Hamamatsu Corp, Japan) and morphometric measurements obtained where crypts were aligned along the crypt axis, as in Figure S1.


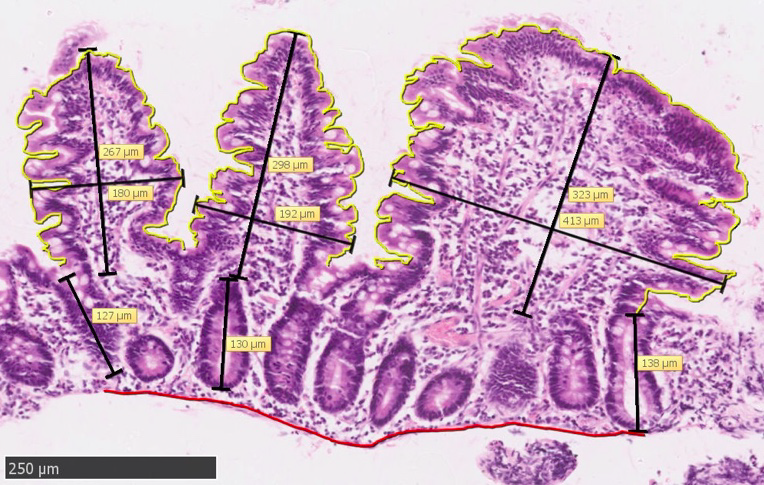


**Figure S1** Morphometric measurements showing villus height, crypt depth, and villus width (all in black), and epithelial surface area as estimated by the villus perimeter (in yellow) in relation to length of muscularis mucosae (in red). Villus cross-sectional area (not shown for clarity) was measured as the area enclosed by the yellow outlines.

**Immunohistochemistry**

Slides were baked for 90 minutes at 60°C, deparaffinised by three washes in xylene (15 minutes each), transferred to 100% ethanol, and rehydrated through graded ethanol solutions. Antigen retrieval was performed for 40 minutes at 95°C using Target Retrieval Solution S2367 (Agilent Technologies). Sections were then washed thrice in TBS (150mM NaCl, 50mM Tris, pH 7.5) with 0.1% tween-20, incubated for 20 min in 50mM NH_4_Cl in TBS, washed thrice with TBS, and incubated in blocking solution (5% BSA, 10% normal donkey serum, 10% normal mouse serum in TBS) with 0.3M glycine to quench tissue autofluorescence. Slides were then incubated with 10 µg/ml rabbit anti-claudin-4 (Abcam, catalogue ab53156, lot GR81356) and 3 µg /ml mouse anti-E-cadherin clone M168 (Abcam, catalogue Ab76055, lot GR216035-3) diluted in blocking solution for 24 hours at 4°C. After three washes in TBS with 0.1% tween-20 and two washes in TBS, a secondary antibody cocktail containing 1.5 µg/ml AlexaFluor 488 AffiniPure donkey anti-mouse IgG (Jackson ImmunoResearch, catalogue 715-545-151, lot 118237), 1.5 µg /ml AlexaFluor 594 AffiniPure F(ab’)2 fragment donkey anti-rabbit IgG (Jackson ImmunoResearch, catalogue 711-586-152, lot 114063), and 1 µg/ml Hoechst 33342 (Thermo Fisher Scientific) for 2 hours at room temperature. After six washes in TBS with 0.1% tween-20, slides were dipped in water and coverslipped using ProLong Gold antifade (Thermo Fisher Scientific). Imaging used an Axioplan 2 microscope equipped with a 20X/NA 0.8 and 63x/ NA 1.4 oil Plan-Apochromat objectives (Carl Zeiss), CoolSnapHQ camera (Photometrics), and single channel ET filter cubes (Chroma). Image z-stacks were acquired using Metamorph 7.8 (Molecular Devices) using matched exposures for each channel and objective pair. Post-acquisition, images were deconvoluted for 10 iterations using Autoquant X3 (Media Cybernetics). Single plans were selected, combined and scaled using MetaMorph and Photoshop CS6 (Adobe Systems).

**PCR amplification of bacterial 16S rDNA**

A primer pair of 16S F519 5'- CAGCAGCCGCGGTAATAC -3' and 16S R785 5'- TGGACTACCAGGGTATCTAATCC -3' was used. Real-time quantitative PCR mix consisted of 2 x Qiagen QuantiTect Probe PCR kit (Qiagen, Hilden, Germany), 0.4 μl of a mixture of both forward and reverse primers at a final concentration of 0.2 mM , 5 μl of DNA and 4.6µl endotoxin-free water to a final volume of 20μl. PCR was performed using Rotor gene 6000 (Corbett, Australia). The amplification reaction profile consisted of an initial denaturation at 95°C for 5 minutes followed by a 40 cycles at 95°C for 15s, 60°C at 20s and 72°C at 45s. For the specific identification of 16S rRNA gene, a melt-curve analysis was conducted by heating PCR products from 65°C to 99°C with continuous acquisition and products were subjected to a ramping temperature of 0.2°C/s. Positive amplification was declared if fluorescence crossed the Ct at 31 cycles or fewer.

**Comparison of morphometric measurements with historical data**

Penna et al^1^ measured crypts and villi in 24 British children with normal biopsies: mean (SD) VH was 332 (45) μm, CD was 169 (28) μm and the VH:CD ratio was 2.00 (0.35). Campbell et al^2^ provided remarkably similar data from a different group of British children: median (SD) VH 355 (35) μm, CD 170 (20) μm and VH:CD 2.1 (0.3). Cook et al^3^ found similar values of VH (median 321 μm, range 271-359 μm) in Ugandan children who had had kwashiorkor 4 years previously and therefore constitute one of the few extant normal ranges for African children. In severely malnourished children VH is consistently reduced: in Gabon^4^, mean VH was 218 μm (SD 43) and CD was 154 (17) μm in 13 children with SAM. However, in 38 children with malnutrition in The Gambia median VH was 243 μm (SD 68) and CD was 278 (69) μm^2^ which is evidence of a markedly hyperplastic mucosal change not seen in the Gabonese children^4^.

1 Penna FJ, Hill ID, Kingston D, Robertson K, Slavin G, Shiner M. Jejunal mucosal morphometry in children with and without gut symptoms and in normal adults. *J Clin Path* 1981; **34**: 386-392.

2 Campbell DI, Murch SH, Elia M, et al. Chronic T cell-mediated enteropathy in rural west African children: relationship with nutritional status and small bowel function. *Pediatr Res* 2003; **54**: 306-11.

3 Cook GC, Lee FD. The jejunum after kwashiorkor. Lancet 1966; ii: 1263-1267.

4 Gendrel D, Gahouma D, Ngou-Milama E, Nardou M, Chamlian A, Philippe E. Anomalies de la muqueuse jejunale et malnutrition protein-calorique chez le nourrisson en Afrique equatorial. *Ann Pediatr* 1984; **31**: 871-876.
